# Identifying featured indels associated with SARS-CoV-2 fitness

**DOI:** 10.1128/spectrum.02269-23

**Published:** 2023-09-12

**Authors:** Xiang Li, Hongliang Yan, Gary Wong, Wanli Ouyang, Jie Cui

**Affiliations:** 1 CAS Key Laboratory of Molecular Virology & Immunology, Shanghai Institute of Immunity and Infection, Chinese Academy of Sciences, Shanghai, China; 2 AI for Science, Shanghai Artificial Intelligence Laboratory, Shanghai, China; 3 University of Chinese Academy of Sciences, Beijing, China; Thomas Jefferson University, Philadelphia, Pennsylvania, USA

**Keywords:** SARS-CoV-2, featured indels, machine learning, genomic epidemiology, evolution

## Abstract

As an RNA virus, severe acute respiratory coronavirus 2 (SARS-CoV-2) is known for frequent substitution mutations, and substitutions in important genome regions are often associated with viral fitness. However, whether indel mutations are related to viral fitness is generally ignored. Here we developed a computational methodology to investigate indels linked to fitness occurring in over 9 million SARS-CoV-2 genomes. Remarkably, by analyzing 31,642,404 deletion records and 1,981,308 insertion records, our pipeline identified 26,765 deletion types and 21,054 insertion types and discovered 65 indel types with a significant association with Pango lineages. We proposed the concept of featured indels representing the population of specific Pango lineages and variants as substitution mutations and termed these 65 indels as featured indels. The selective pressure of all indel types is assessed using the Bayesian model to explore the importance of indels. Our results exhibited higher selective pressure of indels like substitution mutations, which are important for assessing viral fitness and consistent with previous studies *in vitro*. Evaluation of the growth rate of each viral lineage indicated that indels play key roles in SARS-CoV-2 evolution and deserve more attention as substitution mutations.

**IMPORTANCE:**

The fitness of indels in pathogen genome evolution has rarely been studied. We developed a computational methodology to investigate the severe acute respiratory coronavirus 2 genomes and analyze over 33 million records of indels systematically, ultimately proposing the concept of featured indels that can represent specific Pango lineages and identifying 65 featured indels. Machine learning model based on Bayesian inference and viral lineage growth rate evaluation suggests that these featured indels exhibit selection pressure comparable to replacement mutations. In conclusion, indels are not negligible for evaluating viral fitness.

## INTRODUCTION

Recurring flare-ups of severe acute respiratory coronavirus 2 (SARS-CoV-2) have caused substantial obstacles to the functioning of society worldwide. The continuing evolution of SARS-CoV-2 has resulted in the accumulation of several significant substitution mutations, such as L452R, N501Y, and D614G in the spike protein, which is crucial for the capacity of viral transmission and immune evasion (1 – 3). The binding between spike protein and cell receptor is affected by several factors, not only substitution mutations (4 – 8). Currently, genomic and phylogenetic investigations have mainly focused on substitution mutations (9). However, indels, which are deletions and insertions, have long been overlooked because they are more challenging to study than substitution mutations (10).

Although less studied, previous research has reported the importance of some indels; 33 nucleotide deletions from Nsp1 in *ORF1ab* indicated a lower interferon beta (IFN-β) response (11). Another 9-nucleotide deletion in Nsp1 resulted in the deletion of three amino acids (KSF), which may affect the structure of the C-terminus of Nsp1 (12). Other deletions in *ORF1ab* were also reported occasionally, such as the deletion of Asp 268 in Nsp2 and the deletion 106–109 in Nsp6 (13, 14). The frequency of deletions in accessory genes exceeded expectations, in addition to deletions in structural and nonstructural proteins. A 34-nucleotide deletion in *ORF6* caused the upregulation of nine genes in the nuclear factor kappa B (NF-κB) pathway (15). Other deletions occurring in accessory genes seemed to have no clear functions, such as the deletion of four nucleotides overlapping the stop codon of *ORF6* with the initiation codon of *ORF7a* (16). The insertion of ins214EPE in spike’s N-terminal domain (NTD) was widely distributed along with the spread of the Omicron Variant of Concern (VOC). Ins214EPE has only been found in the lineages of the Omicron variant, and its presence was thought to result from template switching (17). Research on the NTD of the *Spike* gene reported a single recurrent insertion region, which contained over 49 types of insertions in this region (18). Since the inserted nucleotides could not appear without the reference sequence, some studies considered these insertions host derived, which may be caused by template switching or polymerase slippage (19, 20).

Research on the locations of indels revealed the reasons for the generation caused by template switching (21). A previous study detected several recurrent deletion/insertion types and demonstrated the relationship between indels and variants (22). The deletion of H69/V70 in the spike protein of the Alpha variant was linked to an increase in immune escape (23). Another deletion, Y144, from the spike protein changed the pocket structure of the NTD and decreased the affinity of NTD binding to neutralizing antibodies (nAbs) (24, 25).

Although the fitness of substitution mutations has been studied and computational efficiency was evaluated based on Bayesian modeling, the fitness of indels has yet to be investigated (26, 27). The selection coefficient, representing the selective pressure of some deletions, was inferred, and the relevance of deletions was highlighted via the Bayesian inference approach based on maximum *a posteriori* (MAP) (27). However, frequent occurrences of indels must be systematically presented and investigated. In addition, the employed MAP estimator assumes all mutations as non-neutral, which is unattractive and improved by Bayesian Viral Allele Selection (BVAS) model (28).

In this study, we collected over 9 million SARS-CoV-2 genomes and systemically analyzed indels that occurred in these genomes. We identified several indels in millions of sequences and proposed the concept of featured indels representing the population of variants and Pango lineages. We also proposed to refine Pango lineages with indel information and computed the fitness of indels. Both the growth rates of clades and the selection coefficient of mutations were assessed more accurately with indels.

## RESULTS

### Massive indels occurred in SARS-CoV-2 genomes

We first downloaded genomes and corresponding metadata of SARS-CoV-2 from the Global Initiative on Sharing All Influenza Data website (GISAID, https://www.gisaid.org/) and carried out quality control on genomes to remove redundant sequences that do not meet the requirements; 9,149,680 genomic sequences passed the screening and were aligned to the reference sequence (NC_045512.2). A SAM file was generated during this process, and indels were then extracted from the generated SAM file. To make the analysis clear, we termed each detected deletion or insertion in a concrete sequence as an indel record here. We defined indels with the same start site and length and the same sequence for insertions as one type of indel. The indel types were termed with Del_start site_length for deletions and Ins_start site_length_sequence for insertions. Indels whose length is an integer multiple of 3 were considered non-frameshift indel types, and the others as frameshift indel types.

**Fig 1:**
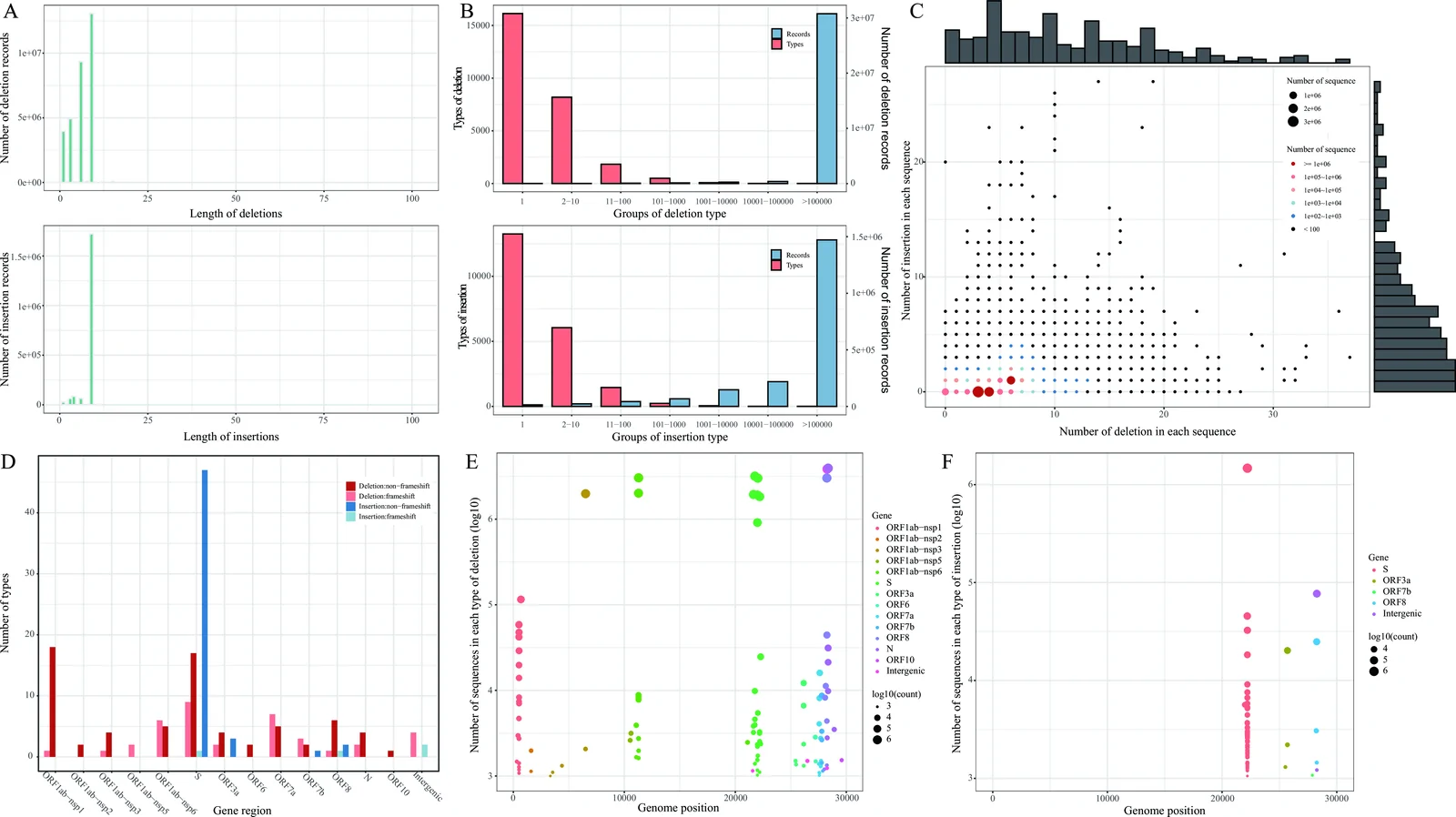
(A) The number of indel records about different lengths of indels. The figure shows only the length of indel less than 100 nucleotides. (B) The abscissa represents seven groups of indel types, the vertical axis represents the sum of indel types on the left and the sum of indel records among seven groups on the right. (C) The figure shows the number of sequences that contain several indel types. The color and size of the circle represent the number of sequences with the corresponding number of indels. (D) The number of indel types in genes and intergenic regions. Only indel types whose number of sequences over 1,000 were counted here. (E) The point indicates the concrete position and the number of each deletion type. The color indicated the gene to which each deletion type belongs. (F) The point indicates the concrete position and the number of each insertion type. The color indicated the gene to which each insertion type belongs.

In total, 31,642,407 deletion records and 1,981,308 insertion records were detected in 9,149,680 filtered genomic sequences after removing indel records in 5′ and 3′ untranslated regions (UTR), and there were 26,765 different types of deletions and 21,054 different types of insertions (Table S1). Length of deletions varied from 1 nucleotide to 708 nucleotides, and the length of insertions varied from 1 nucleotide to 697 nucleotides, while longer indels appeared less frequently as expected (Table S2). However, several lengths, like deletions of 1, 3, 6, 9 nucleotides and insertion of 9 nucleotides, are extraordinarily more than others (Fig. 1A). To better study the overall distribution of indel, we separated indels into seven groups according to the number of indel records in each type of indels. There are a total of seven groups, which are occurrence 1, 2–10, 11–100, 101–1,000, 1,001–10,000, and more than 10,000. More than 90% of indel types belong to groups that occurred in fewer than 10 sequences, and the percentages of records in these groups are 0.15% in deletions and 1.81% in insertions (Fig. 1B; Table 1). However, in groups of types that occurred in more than 1,000 sequences, indel records accounted for 99.19% and 92.70% in deletion and insertion, respectively. As a significant proportion of indels correspond to a limited number of types, we aimed to investigate whether these indels originated from the same genomic sequence or were randomly distributed.

**TABLE 1:** Types and number of indel records in about seven groups^*a*^

Group	Types of deletion	Percentage (%) of deletion types	Number of deletion records	Percentage (%) of deletion records
1	16,133	60.2765	16,133	0.0510
2–10	8,196	30.6221	28,667	0.0906
11–100	1,829	6.8336	55,594	0.1757
101–1,000	500	1.8681	155,490	0.4914
1,001–10,000	81	0.3026	245,984	0.7774
10,001–100,000	13	0.0486	372,252	1.1764
>100,000	13	0.0486	30,768,287	97.2375

Group	Types of insertion	Percentage (%) of insertion types	Number of insertion records	Percentage (%) of insertion records
1	13,261	62.9857	13,261	0.6693
2–10	6,054	28.7546	22,600	1.1407
11–100	1,449	6.8823	42,408	2.1404
101–1,000	233	1.1067	66,446	3.3536
1,001–10,000	50	0.2375	146,477	7.3929
10,001–100,000	6	0.0285	218,402	11.0231
>100,000	1	0.0047	1,471,714	74.2799

^*a*^ The groups were divided based on the number of sequences for each indel type. Only indel types that meet the requirement of the group will be counted. The number of indel records is the sum of all indel records in this group.

We collected information about the occurrence of indels in concrete sequences and found that the distribution of indels had a particularly concentrated tendency. The number of sequences with three types of deletion was over 3 million (Fig. 1C). The number of sequences with four types of deletion and sequences without indels exceeded 1.9 million. The number of sequences with six types of deletion and one type of insertion and the number of sequences with only one type of deletion also exceeded 1 million. Deletions occurred more commonly than insertions when specific to each sequence. Deletions tended to appear less than seven times, and insertions tended to appear less than one time in a single sequence (Table 2). There also other indels occurring in a single sequence, but these were far less than mentioned (Table S3). Notably, indels are not randomly distributed but have strong regularity.

**TABLE 2:** The sequences with a specific number of deletions and insertions^*a*^

Number of deletion	Number of insertion	Number of sequences	Groups
3	0	3,290,657	≥1e + 06
4	0	2,144,942	≥1e + 06
6	1	1,433,054	≥1e + 06
0	0	807,832	1e + 05–1e + 06
5	0	367,499	1e + 05–1e + 06
2	0	284,933	1e + 05–1e + 06
5	1	203,541	1e + 05–1e + 06
6	0	167,943	1e + 05–1e + 06
1	0	117,815	1e + 05–1e + 06
1	1	88,713	1e + 04–1e + 05
7	1	77,429	1e + 04–1e + 05
4	1	61,364	1e + 04–1e + 05
3	1	35,795	1e + 04–1e + 05
0	1	14,306	1e + 04–1e + 05
6	2	11,235	1e + 04–1e + 05
7	0	9,766	1e + 03–1e + 04
2	1	8,756	1e + 03–1e + 04
8	1	7,114	1e + 03–1e + 04
7	2	3,452	1e + 03–1e + 04
5	2	1,995	1e + 03–1e + 04
8	0	1,455	1e + 03–1e + 04
4	2	1,015	1e + 03–1e + 04

^*a*^ Only groups with over 1,000 sequences are presented here.

Indel types varied among genes. The *Spike gene* had the most diverse indel types, followed by *ORF1ab-nsp3* and *ORF7a* (Fig. S1). The number of frameshift deletion types was more than non-frameshift generally and the same for frameshift insertion, except for non-frameshift insertion in the *Spike gene* (Table S4). However, it became the opposite when we focused on indels whose sequences exceeded 1,000. Indels in the *Spike gene* were also the most diverse, especially for non-frameshift insertions (Fig. 1D). The number of non-frameshift deletion types was higher than frameshift deletions in most genes (Table 3). Although there were so many indel types in these genes, most occurred in less than 1,000 sequences (Fig. S2 and S3). Only 12 types of deletion and 1 type of insertion occurred in over 1 million sequences. It is worth noting that one of those deletions occurred in the intergenic region (Fig. 1E and F). The indel types distribution in over 1,000 sequences tends to cluster in some specific gene regions. The deletion types over 1,000 sequences clustered more in NTD of the *Spike* gene, *ORF1ab-nsp1*, *ORF1ab-nsp6,* and *N*, while insertion types over 1,000 sequences tended to cluster in NTD of the *Spike* gene and accessory genes.

**TABLE 3:** The number of indel types with frameshift or non-frameshift in different genes^*a*^

Gene	FS/NFS	Deletion	Insertion
ORF1ab-nsp1	Frameshift	1	0
	Non-frameshift	18	0
ORF1ab-nsp2	Non-frameshift	2	0
ORF1ab-nsp3	Frameshift	1	0
	Non-frameshift	4	0
ORF1ab-nsp5	Frameshift	2	0
ORF1ab-nsp6	Frameshift	6	0
	Non-frameshift	5	0
S	Frameshift	9	1
	Non-frameshift	17	47
ORF3a	Frameshift	2	0
	Non-frameshift	4	3
ORF6	Non-frameshift	2	0
ORF7a	Frameshift	7	0
	Non-frameshift	5	0
ORF7b	Frameshift	3	0
	Non-frameshift	2	1
ORF8	Frameshift	1	1
	Non-frameshift	6	2
N	Frameshift	2	0
	Non-frameshift	4	0
ORF10	Non-frameshift	1	0
Intergenic	Frameshift	4	2

^*a*^ Only indel types that occurred in over 1,000 sequences were counted.

### Characteristic featured indels represented the population of lineages

**Fig 2:**
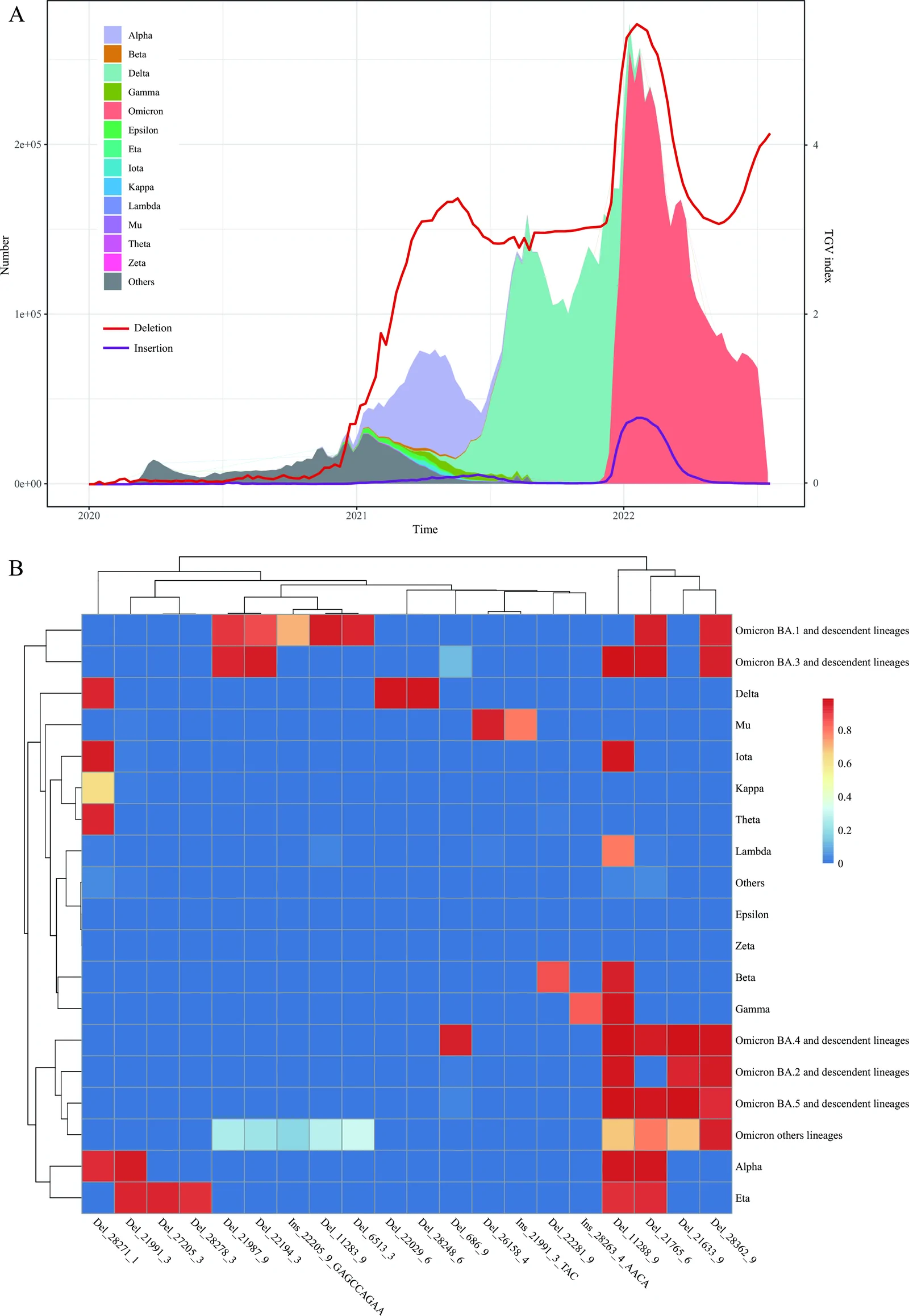
(A) TGV index of deletion and insertion. The stacked areas represent the number of sequences in variants. (B) Heatmap of 20 indel types and variants. The color indicates the ratio of sequences containing indels in the corresponding variant.

We next examined the pattern of indels among circulating SARS-CoV-2 over time. To explore the variation of indel among variants, the temporal genetic variation index (TGV index) of indel was defined here as the ratio of the number of indel records to the number of sequences per week. We tallied the number of deletion and insertion records per week and calculated the TGV indices of deletions and insertions, respectively. The TGV index exhibited a stronger correlation with variants and increased obviously during the Alpha, Delta, and Omicron variants, especially for deletions (Fig. 2A). As the Alpha variant became widespread, the TGV index of deletion increased significantly and even reached 3.3, which meant that the sequence in the Alpha variant had three deletion records on average during this period. The TGV index of deletion fell back when the Delta variant was circulating but increased rapidly again when the Omicron variant became widespread, indicating three deletion records in the Delta variant and 5.5 in the Omicron variant. The TGV deletions index rose again when other Omicron lineages were circulating. The TGV index of insertion exhibited a slight increase in the population when the Gamma variant was circulating but increased rapidly when the Omicron variant was circulating. From the rise of the TGV index, we speculated that an internal connection might exist between indels and the population of SARS-CoV-2 variants.

The Pango lineage is a widely used system for the classification of SARS-CoV-2. To investigate the internal link, we performed intricate computations regarding the ratios of indels, particularly Pango lineages. The ratio of 47,819 indel types in each Pango lineage was then calculated (Table S5). To better understand, only indel types occurred in at least 80% of sequences of Pango lineages were kept, and a heatmap between 626 Pango lineages and 65 filtered indel types was constructed (Table S6). Since these 65 indel types accounted for more than 80% in at least one Pango lineage, we termed these indel types as featured indels because these indel types can be used as a new class of features related to the Pango lineage. Featured indels in the heatmap exhibited strong agglomerative characteristics. Pango lineages belonging to the same variants tended to have the same combination of featured indels, although there was some overlap among these indel types (Fig. S4). For example, it is obvious that seven indel types clustered in lineages BA.1.* and three deletion types clustered in lineages AY.* Since there were hundreds of Pango lineages, it was too complicated to analyze all Pango lineages and indels simultaneously. We recalculated and summarized the proportion of indel types among different variants according to corresponding Pango lineages (Fig. 2B).

Alpha variants composed of B.1.1.7 and Q.* had four deletion types with high proportion which were Del_11288_9, Del_21765_6, Del_21991_3, and Del_28271_1, except for Del_11288_9 in Q.2. Delta variant composed of B.1.617.2 and AY.* had three deletion types, Del_22029_6, Del_28271_1, and Del_28248_6. The proportion of featured deletions varied among lineages in the Delta variant. Although most lineages in the Delta variant exhibited a higher proportion of featured deletions, some in the Delta variant had a lower rate. For example, Del_28271_1 had a rate over 90% in most Delta lineages but lower than 60% in some lineages, such as AY.21, AY.38, AY.77, and AY.112.1 (Fig. S4).

Featured indels in lineages belonging to the Omicron variant exhibited completely different distribution characteristics compared to the Alpha and Delta variants. Six featured deletions, Del_6513_3, Del_11283_9, Del_21765_6, Del_21987_9, Del_22194_3, and Del_28362_9, and one featured insertion, Ins_22205_9_GAGCCAGAA, exhibited higher proportions among BA.1 and descendent lineages. BA.2 and descendent lineages only had three featured deletions, Del_11288_9, Del_21633_9, and Del_28362_9. Five featured deletions, Del_11288_9, Del_21765_6, Del_21987_9, Del_22194_3, and Del_28362_9, were enriched in BA.3 and descendent lineages. In addition, five featured deletions, Del_686_9, Del_11288_9, Del_21633_9, Del_21765_6, and Del_28362_9, were enriched in BA.4 and descendent lineages. Four featured deletions, Del_11288_9, Del_21633_9, Del_21765_6, and Del_28362_9, were enriched in BA.5 and descendent lineages. Although the Omicron variant had many featured indels, several indels were shared among these lineages. Del_28362_9 occurred in all lineages belonging to the Omicron variant. BA.1 and descendent lineages can be divided into one group, and BA.2-BA.5 can be divided into another group according to the distribution of featured indels. The BA.2-BA.5 and descendent lineages shared several featured deletions, such as Del_11288_9, Del_21765_6, and Del_21633_9, which were not shared with BA.1. From the perspective of featured indels, the Omicron variant was similar to a complex group of multiple different variants that had their own featured indels.

Other variants also had specifically featured indels. For example, the Beta variant had Del_11288_9 and Del_22281_9. Gamma variant had Del_11288_9 and Ins_28263_4_AACA. Mu variant had Del_26161_4 and Ins_21991_3_TAC. Iota variant had Del_11288_9 and Del_28271_1. Eta variant had Del_11288_9, Del_21765_6, Del_21991_3, Del_27205_3, and Del_28278_3. Theta variant had Del_28271_1. Although almost every variant had its featured indels, and some were shared among different variants. Featured deletion Del_11288_9 appeared in almost all variants except for the Delta, Mu, and BA.1.* belonging to the Omicron variant. However, BA.1.* had another featured deletion, Del_11283_9 instead, which overlapped with Del_11288_9 and indicated the key function of deletions in *ORF1ab-nsp6*. Besides, almost all variants had at least one featured deletion or featured insertion in the NTD of *Spike*. In summary, the high proportion of featured indels can represent the population of different variants.

### Additional indel information refined Pango lineages

**Fig 3:**
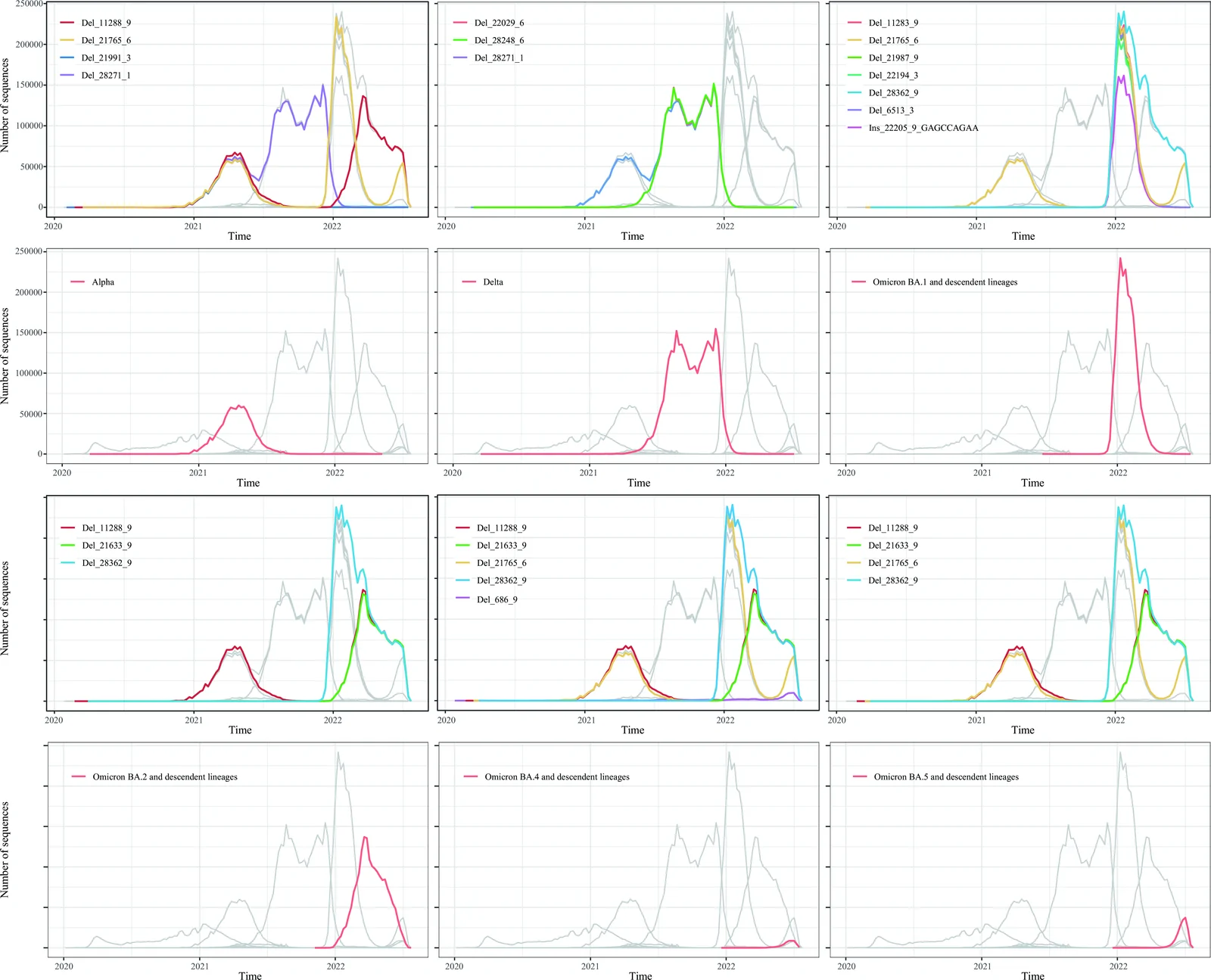
The change in the number of sequences containing corresponding indels with time and the change in the number of sequences in different variants.

Featured indels exhibited higher proportions among variants and Pango lineages. We computed the number of featured indels over time among variants to determine the precise trend of featured indels. Featured indels were strongly correlated with epidemic variations and exhibited a higher degree of consistency among variants (Fig. 3; Fig. S5). Multiple abnormally fluctuating indels are also due to shared featured indels, which exhibited a similar trend when corresponding variants were prevalent.

**Fig 4:**
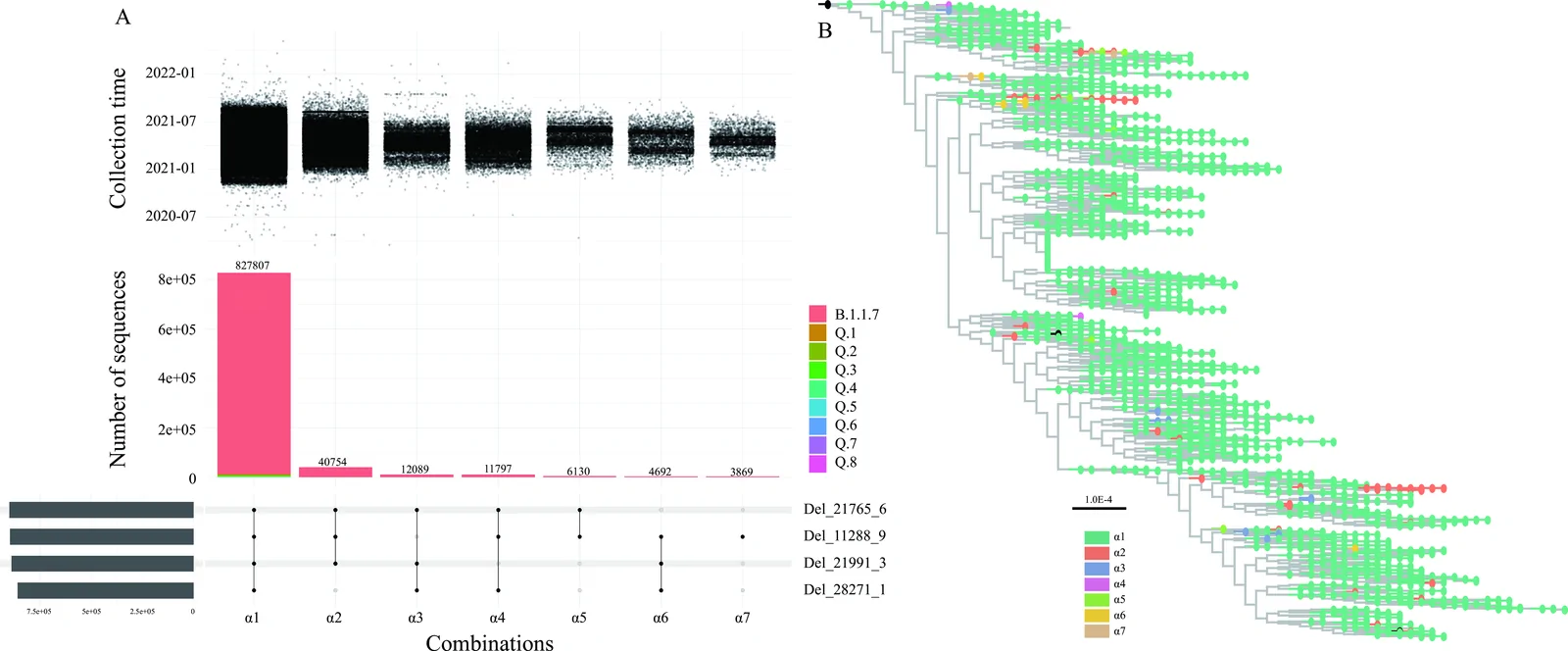
(A) The upset diagram exhibits the combination of four featured deletions and the corresponding number. The color in each combination means the Pango lineages of the Alpha variant. The upper panel plotted dots of collection dates corresponding to sequences in each combination. (B) The phylogenetic tree of lineage Q.1. The color represents the combination of α1–α7.

Although featured indels in different variants had high consistency, the quantities of featured indels among specific variants were not mutually equal. The unequal number implied several potential combinations of featured indels in concrete sequences, which means that not all sequences in specific variants simultaneously possessed all featured indels. To investigate the composition, we tallied the number, collection dates, and continents of combinations of featured indels. Most sequences in the Alpha variant mainly existed in the first seven combinations (Fig. S6). The combination α1 of four featured deletions Del_11288_9, Del_21765_6, Del_21991_3, and Del_28271_1 had the highest number with 827,807 (89.75% of Alpha variant) sequences, far more than other combinations, such as the combination α2 of Del_11288_9, Del_21765_6, and Del_21991_3 (40,754 sequences, 4.42%) and the combination α3 of Del_21765_6, Del_21991_3, and Del_28271_1 (12,089 sequences, 1.31%) in the Alpha variant (Fig. 4A, lower panel). The summary of the remained combinations was far less than the combination α1. As for time, the collection dates of the combination α1 existed throughout the population of the Alpha variant, while other combinations appeared later and disappeared earlier compared to the first combination (Fig. 4A, upper panel). As for location, to exclude the potential influence of local transmission, we calculated the proportion of each combination on six continents. Six continents all had similar combinations ratios, indicating a little effect on local transmission and spontaneous repair of featured indels (Table 4; Table S7). All combinations of four feature deletions appeared in lineages B.1.1.7 and Q.1–8 regardless of how the featured indels combined, implying that the combination of featured indels between different lineages of the same variant had inherent regularity, which was ignored during previous research. To investigate the combinations of featured indels in phylogeny, we chose lineage Q.1 as an example and checked whether these combinations clustered. The combinations α2–α7 did not cluster in a single branch and were distributed randomly (Fig. 4B).

**TABLE 4:** The ratio of seven combinations of Alpha variants on six continents

Combination	Africa (%)	Asia (%)	Europe (%)	North America (%)	Oceania (%)	South America (%)
α1	83.40	96.25	89.23	95.97	90.62	91.29
α2	14.49	2.98	5.37	2.18	4.24	5.54
α3	0.27	0.22	1.84	0.16	0.15	0.04
α4	1.35	0.33	1.57	0.78	3.48	1.42
α5	0.11	0.05	0.85	0.35	1.36	0.66
α6	0.38	0.16	0.65	0.24	0.15	0.69
α7	0.00	0.01	0.50	0.33	0.00	0.36

**Fig 5:**
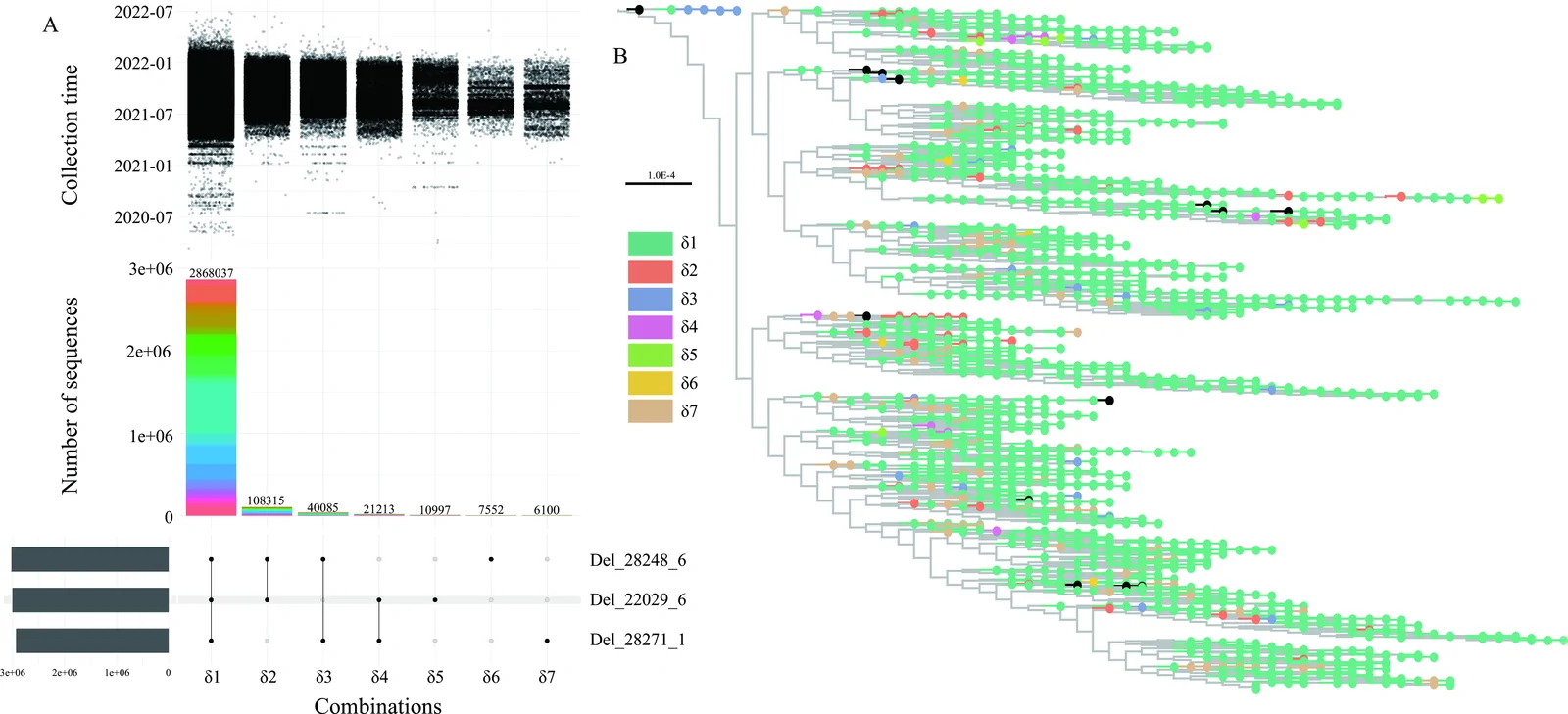
(A) The upset diagram exhibits the combination of three featured deletions and the corresponding number. The color in each combination means the Pango lineages of the Delta variant. The upper panel plotted dots of collection dates corresponding to sequences in each combination. (B) The phylogenetic tree of lineage AY.13. The color represents the combination of δ1–δ7.

Featured indels are similar to the Alpha variant in the Delta variant. The featured deletions, Del_22029_6, Del_28248_6, and Del_28271_1, had seven different combinations. Since more than 200 Pango lineages belong to the Delta variant, the composition of Pango lineages in seven combinations exhibited was complex. The combinations of featured indels simultaneously existed in different lineages (Fig. 5A, lower panel, Fig. S7). Combination δ1 still had the widest range of collection dates, and other combinations also appeared later and disappeared early than combination δ1 (Fig. 5A, upper panel). Combination δ1 of all three featured indels occupied the highest proportion in six continents, while combination δ2 exhibited a higher proportion in Africa than in other continents (Table 5; Table S8). Sequences in lineage AY.13 were selected to construct the phylogenetic tree, and sequences in different combinations were plotted with different colors. The disappearance of featured deletions was also random (Fig. 5C).

**TABLE 5:** The ratio of seven combinations of Delta variants on six continents

Continent	Africa (%)	Asia (%)	Europe (%)	North America (%)	Oceania (%)	South America (%)
δ1	76.61	96.52	92.95	94.52	89.90	93.91
δ2	17.90	0.72	4.64	2.03	9.30	4.60
δ3	1.73	1.05	1.29	1.41	0.44	0.99
δ4	0.44	1.02	0.33	1.20	0.31	0.19
δ5	2.31	0.13	0.44	0.27	0.05	0.12
δ6	0.96	0.23	0.29	0.19	0.00	0.09
δ7	0.06	0.33	0.07	0.38	0.00	0.11

**Fig 6:**
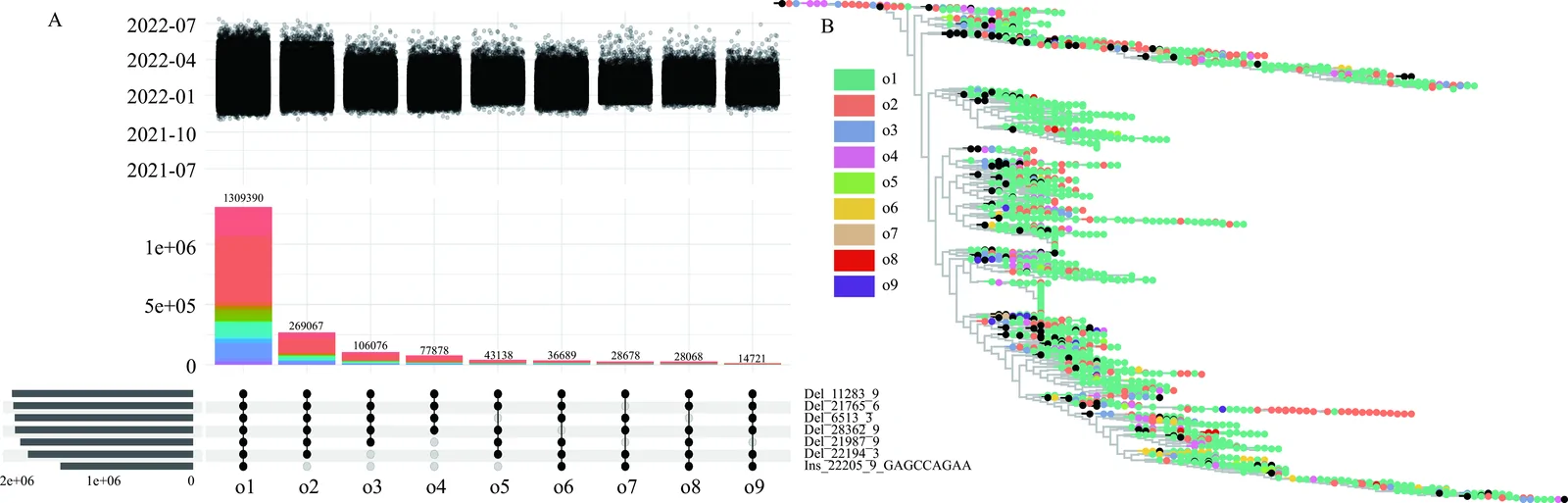
(A) The upset diagram exhibits the combination of six featured deletions and one featured insertion and the corresponding number. The color in each combination means the Pango lineages belonging to BA.1 and the descendent lineages of the Omicron variant. The upper panel plotted dots of collection dates corresponding to sequences in each combination. (B) The phylogenetic tree of lineage BA.1.21. The color represents the combination of o1–o9.

Combinations of featured indels were more complex in Omicron Variant. Seven featured indels in BA.1.* had more than 100 combinations (Fig. S8). Similar to Alpha and Delta variants, combinations o1 with seven featured indels had the largest number, and the number of other combinations was small when a focused number of over 10,000 sequences (Fig. 6A, lower panel). But the collection dates of BA.1.* exhibited a mild trend of late appearance and early disappearance (Fig. 6A, upper panel). On different continents, these combinations also maintained roughly the same proportions (Table 6; Table S9). The distribution of different combinations was also similar to the Alpha and Delta variants based on the sample phylogenetic tree of BA.1.21 (Fig. 6B).

**TABLE 6:** The ratio of nine combinations of BA.1 and descendent lineages belonging to the Omicron variant on six continents

Continent	Africa (%)	Asia (%)	Europe (%)	North America (%)	Oceania (%)	South America (%)
o1	63.49	84.16	71.97	62.51	69.52	60.14
o2	15.41	8.28	11.39	17.94	8.34	18.07
o3	4.87	1.55	3.84	8.19	1.11	6.67
o4	6.98	1.90	3.49	4.48	0.18	13.56
o5	2.21	0.55	3.19	1.49	1.31	0.49
o6	4.31	2.24	1.10	2.76	11.36	0.36
o7	0.31	0.27	2.18	0.98	0.00	0.13
o8	1.48	0.70	2.19	0.66	8.10	0.24
o9	0.95	0.35	0.67	0.99	0.08	0.35

Combinations of featured indels in lineages of other Omicron variants demonstrated different collection dates. Few featured indels indicated late and early disappearance, indicating a potential indel-related virus evolution pattern (Fig. S9 to S12). Other variants such as Beta, Eta, Gamma, Iota, and Mu also exhibited similar characteristics for combinations of featured indels and trends of collection dates (Fig. S13 to S17). From the perspective on featured indel combinations, there should be finer divisions inside Pango lineages.

Inspired by combinations of featured indels, we considered a more detailed clustering method that added indel information to existing lineages. We clustered 1,846 Pango lineages into 3,000 clusters with substitution mutations and then refined 1,846 Pango lineages into 3,092 clusters by adding additional indel information in addition to substitution mutations. Most refined clades were the same as clades refined with only substitution mutations, while some clades whose sequences were assigned to a single clade were divided into two or more clades (Table S10). In-depth analysis showed that several refined clusters based on substitution mutations were separated into two or more new clusters based on substitution mutations and featured indels (Table 7). Adding indel information was beneficial for obtaining finer clusters.

**TABLE 7:** Six examples of refined clades^*a*^

Clade0	Clade1	Clade2	Pango lineage	Mutation 1	Mutation 2	Fitness0	Fitness1	Fitness2
Clade1-0	Clade1-1	Clade1-2	B.1.1.7		ORF1a:Del_11288_9	1.9773	2.0171	2.0187
Clade2-0	Clade2-1	Clade2-2	B.1.1.7		Del_21765_6	1.8849	1.9263	1.9263
Clade3-0	Clade3-1	Clade3-2	AY.122		Intergenic:Del_28271_1	3.3917	3.4032	3.4428
Clade4-0	Clade4-1	Clade4-2	AY.100		Intergenic:Del_28271_1	3.3928	3.4112	3.4508
Clade5-0	Clade5-1	Clade5-2	BA.1.1		S:Del_21765_6	6.0561	6.1001	6.1000
Clade6-0	Clade6-1	Clade6-2	BA.2	S:Del_21633_9		7.1535	7.3511	7.1952

^*a*^ Clade0 indicates the original clade refined only with substitution mutations. Clade0 was divided into Clade1 and Clade2 based on indels. Pango lineage indicates the sequences belonging to. Mutation 1 and Mutation 2 correspond to Clade1 and Clade2, respectively. Fitness0, Fitness1, and Fitness2 correspond to fitness of Clade0, Clade1, and Clade2, respectively.

### Indels have a non-negligible impact on viral fitness

**Fig 7:**
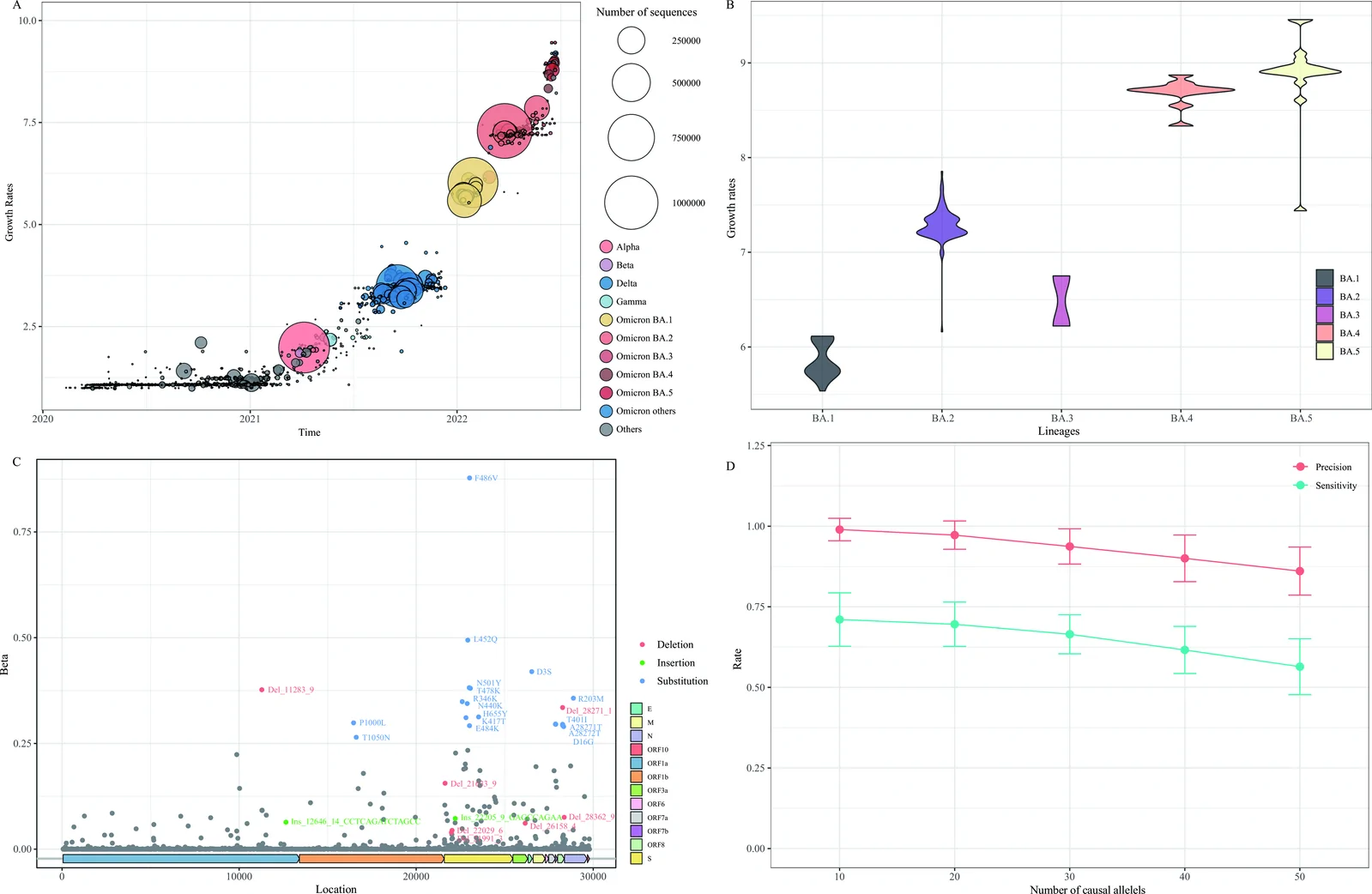
(A) The figure exhibits the growth rates of all Pango lineages estimated with substitution and indel mutations. The color indicates the variants. The size of the circle indicates the number of sequences of a Pango lineage. (B) The violin chart indicates the growth rate distribution among lineages belonging to BA.1–BA.5. (C) The figure exhibits the selection coefficient of different mutations estimated with substitution and indel mutations. Red, green, and blue points indicate the selection coefficient of deletion, insertion, and substitution, respectively. Beta represents the selection coefficient. (D) Red and blue lines indicate the simulation’s precision and sensitivity in 10–50 non-neutral mutations.

BVAS has been shown to evaluate the growth rates of refined clusters and the selection coefficient of substitution mutations effectively and efficiently. However, due to the difficulties of including indels in clusters, BVAS focused more on substitution mutation information conventionally. We employed revised clades with substitution mutations and additional indel information as input for BVAS to better assess viral fitness. Here we used growth rates to represent the viral fitness, equivalent to a multiple of the reproductive capacity of the most original strain numerically. The results of the fitness estimation using indel information were essentially similar but more accurate than those using only substitution mutations (Fig. 7A; Fig. S18, Tables S11 and S12). With lineages BA.5.2.3 and BA.5.2.2 growing at a pace approximately 9.46 times higher than the original strain, lineages belonging to the Omicron variant still demonstrated greater viral fitness than other variants (Table 8). The top 20 lineages in terms of growth rates were all Omicron variants, and the majority had only recently formed in very contagious lineages, which was consistent with the pandemic trend. The growth rates of lineages in the Omicron variant varied from 5.5 to 9.5, a larger range than other variants. The wide range and local clustering of growth rates in BA.1–BA.5 lineages of the Omicron variant supported the separation of the Omicron variant (Fig. 7B).

**TABLE 8:** The growth rates of the top 20 Pango lineages

Rank	Lineage	Growth rate	Std
1	BA.5.2.3	9.456159138	0.375967123
2	BA.5.2.2	9.454890179	0.375551289
3	BF.5	9.193990358	0.372186209
4	BF.4	9.192086734	0.37183592
5	BF.6	9.191671864	0.371826775
6	BF.2	9.191349775	0.371726312
7	BA.5.2.4	9.099300494	0.38848994
8	BA.5.2	9.030993689	0.369713811
9	BA.5.2.1	8.961810123	0.373541511
10	BE.1.1	8.958672664	0.373174154
11	BA.5.3.2	8.952308735	0.380195692
12	BA.5.6	8.950152409	0.376489933
13	BF.1	8.94664629	0.38852285
14	BF.1.1	8.921523457	0.382518495
15	BA.5.3.4	8.917753027	0.37102558
16	BA.5.3.1	8.90576915	0.367579344
17	BA.5.1.2	8.901856067	0.367626747
18	BA.5.1.4	8.901770602	0.367392577
19	BA.5.3.3	8.901415205	0.367331075
20	BA.5.1.1	8.896720964	0.368106318

Growth rates of the Delta variant, ranging from 2.8 to 4.6, were lower than that of the Omicron variant but higher than the Alpha variant. The growth rates of several Pango lineages in the Delta variant tended to congregate in particular places and lacked polycentricity and decentralization. The order of growth rate is Omicron > Delta > Alpha, consistent with global viral transmission. In addition, we contrasted the growth rates of refined clusters that had and lacked indel information with concrete lineages showing the slightest variation (Fig. S18).

The growth rates of specific lineages depend on mutations, and BVAS can estimate the selection coefficient of mutations (Fig. S19). Featured indels also had higher selection coefficients as substitution mutations after adding indels for modified BVAS (Fig. 7C). Substitution mutations F486V in the *Spike* gene had the highest selection coefficient score of 0.88, followed by L452Q, T478K, and N501Y in *Spike* gene and D3S in *Membrane* gene (Tables S13 and S14). Featured deletions Del_11283_9 in *ORF1ab-nsp6* had a selection coefficient score of 0.38, the highest in all deletion types and higher than most other mutations (Fig. 7C; Table 9). Featured deletions Del_28271_1 located between *ORF8* and *Nucleocapsid* also exhibited a higher selection coefficient similar to Del_11283_9, followed by Del_21633_9 in the *Spike* gene. The higher selection coefficient of these three featured deletions suggested a significant contribution to viral growth rates. The three highest-scoring featured deletions corresponded exactly to three widespread variants. The distribution of Del_28271_1 in Alpha and Delta variants, Del_11283_9 in Omicron BA.1*, and Del_21633_9 in Omicron BA.2-BA.5* may be an essential factor in their popularity. Other featured deletions also had stronger selection coefficients than we expected, which implied equal significance for substitution mutations and indels (Fig. 7C). Aside from top-ranked deletions, the remaining indels had a relatively lower selection coefficient (Table S13), which indicates reduced viral fitness.

**TABLE 9:** The selection coefficient of top 10 indels^*a*^

Gene	Mutation	Location	PIP	Beta
ORF1a	Del_11283_9	11,283	0.97	0.38
Intergenic	Del_28271_1	28,271	1.00	0.33
S	Del_21633_9	21,633	0.96	0.16
ORF9b	Del_28362_9	28,362	0.30	0.08
S	Ins_22205_9_GAGCCAGAA	22,205	0.30	0.07
ORF1a	Ins_12646_14_CCTCAGATCTAGCC	12,646	0.26	0.06
ORF3a	Del_26158_4	26,158	0.31	0.06
S	Del_22029_6	22,029	0.25	0.04
S	Del_21991_3	21,991	0.25	0.04
S	Del_21987_9	21,987	0.05	0.01

^*a*^ PIP indicates the posterior inclusion probability. Beta represents the selection coefficient.

### Further analysis of the top-ranked indels

Although we noticed the importance of featured indels, indels were previously far less studied than substitution mutations. Del_11283_9, located in *ORF1ab-nsp6,* had the highest selection coefficient of 0.38, which was higher than most substitution mutations and resulted in the deletion of “LSG” in *ORF1ab-nsp6* (Table 9). Del_11283_9 was a featured deletion representing the population of lineages BA.1* belonging to the Omicron variant. While another featured deletion Del_11288_9, which represents the population of BA.2–BA.5* belonging to the Omicron variant and Alpha, Beta, Gamma, and Iota variant, had a selection coefficient of 0.006 and much smaller than Del_11283_9 (Fig. 2B; Table S13). Featured deletion, Del_28271_1, was the most important feature in the Alpha and Delta variants. Since Del_28271_1 only deleted a single nucleotide and was in the intergenic region between *ORF8* and *Nucleocapsid*, Del_28271_1 has been neglected for a long time. However, Del_28271_1 occurred in over 3 million sequences and had a selection coefficient of 0.33, which was more important and useful than expected. Research on Del_28271_1 demonstrated that sequences with A deletion at site 28,271 had higher transmissibility than those without this deletion, which may be caused by the change in the core Kozak site in *Nucleocapsid* gene (29, 30). Del_21633_9 had a selection coefficient of 0.16 and represented the population of BA.2–BA.5* lineages belonging to the Omicron variant. Other top-ranked featured indels with positive selection coefficients were also reported sometimes. Del_28362_9 was undetectable by PCR since most PCR depended on primers binding to the *ORF1ab* and *N* genes (31). Del_21991_3 was well-studied and featured deletions of the Alpha variant and resulted in the deletion of Y144, which has been reported to affect the transmission ability of SARS-CoV-2. Ins_22205_9_GAGCCAGAA resulted in ins214EPE in *Spike*, which may also play a key role in viral evolution.

## DISCUSSION

The significance of indels, a long-ignored type of mutation, has been underestimated in estimating viral fitness. Few systematic studies focused on the relationship between indels and the pandemic, and most focused on substitutions. Our study systematically identified indels in genomic sequences and discovered the relationship between indels and circulating variants. We proposed the definition of featured indels which can represent the population of Pango lineages and variants and found the internal connection among different combinations of featured indels with variants. Then we modified BVAS, to assess the indel and evaluate the significance of indels and growth rates of different lineages. We found that several indels may contribute to the transmission of SARS-CoV-2.

Indels on different genes may affect transmissibility in various ways. Indels in structural genes may affect the infection by changing the structure of spike protein. As mentioned in the introduction section, it has been proved that H69/V70 and Y144 significantly impacted infection by changing the structure of NTD in *Spike*, which corresponds to featured deletions Del_21765_6 and Del_21991_3 (23 – 25). Some research also reported that immune escape from neutralizing antibodies was also due to deletions in NTD (14, 32). In our analysis, we found all featured deletions that occurred in the Spike gene were in NTD; this could be potential evidence for the functions of deletions in *Spike*. The featured insertions in Spike were Ins_21991_3_TAC and Ins_22205_9_GAGCCAGAA, and fewer experiments were performed to evaluate the functions. Research reported that Ins_22205_9_GAGCCAGAA might increase transmissibility by enhancing sialic-acid receptor binding (17, 33). Four featured deletions, Del_28278_3, Del_28362_9, Del_28890_12, and Del_28896_3, were in the *Nucleocapsid* gene. Del_28362_9 was reported as N-gene target failure (NGTF) in the detection process while other featured deletions in the *Nucleocapsid* gene were less studied (34). Indels in nonstructural genes may affect the replication of SARS-CoV-2. Several featured indels were located in *ORF1ab,* and most of them were in *ORF1ab-nsp1*, *ORF1ab-nsp3,* and *ORF1ab-nsp6*. Del_11283_9 and Del_11288_9 are located in *ORF1ab-nsp6,* and both occurred in the widespread variants such as Alpha, Delta, and Omicron variants. Indel types in accessory genes were more than other genes. Unlike featured indels in structural and non-structural genes, several featured indels in accessory genes were frameshift. Del_27267_29 (featured deletion of B.1.616), Del_27607_17 (featured deletion of AY.39.1), Del_27897_5 (featured deletion of B.1.625), and Del_27922_35 (featured deletion of B.1.623) located in *ORF6*, *ORF7a*, and *ORF8,* respectively. Indels and substitution mutations in intergenic regions should also be focused. Mutations in intergenic regions are usually ignored since they do not encode proteins. However, Del_28271_1 and SNP: A28271T were two mutations that can influence viral evolution, via changing the structure of core Kozak regions and the expression of *N* and *ORF9b*. No experiments were performed to test the influence of these variations, but the statistics model indicated the significance of the A nucleotide in 28,271 (29, 30).

Combinations of featured indels were interesting discoveries and may be closely related to the viral epidemic. Weng et al. have noticed that variants had a unique combination of recurrent deletion pattern regions (14). In our analysis, almost every variant and most Pango lineages had their combinations of featured indels. And the combinations of featured indels can indicate virus epidemic ability to some extent. We also noticed the existence of diverse combinations of different featured indels. Combinations of featured indels varied among variants, but they all followed the same rule: the combination with all featured indels had the largest number of sequences, and the sum of all the remaining combinations was far less than the combination with all featured indels. In addition, combinations of featured indels had other characteristics. Combinations with all featured indels were the earliest to appear, and the latest to disappear from the collection dates, which is puzzling because the other combinations without some featured indels appeared late, which means that the deleted sequence was “added up” and the inserted sequence was “cutoff” from the genome. Phylogenetic analysis of specific lineages showed that the disappearance of indels did not cluster in a single clade but was distributed randomly, which means the spontaneous disappearance of indels. Generally, the probability of reversion of mutations that have already occurred is quite low. The disappearance of featured deletions at different times, in different locations and randomly distributed on the phylogenetic tree indicated potential co-infection. However, the recurrence of feature indels is worthy of further confirmation and investigation.

There is another possibility for the generation of indel combinations. For example, hitchhiking. Hitchhiking means some mutations change frequency under the selection of another mutation linked with previous mutations and is common in genetic variation. There is also hitchhiking in the evolution of SARS-CoV-2. Linkage disequilibrium was found during early transmission for T8782C(*ORF1ab*) and S84L(*ORF8*) although T8782C was a synonymous mutation and do not change amino acids (35). The combinations of featured indels may be caused by hitchhiking. Not all featured indels occurred in structural genes. Some accessory genes may be obtained because they are linked with those featured indels.

Although featured indels were highly correlated with Pango lineages, the evaluation of indels was not the same as we observed. For example, Del_21765_6 was one of the featured deletions in Alpha, Eta, and Omicron variants. However, the selection coefficient of Del_21765_6 was −0.0000196, which even negatively impact the evolution of SARS-CoV-2 according to the evaluation (Table S12). Other featured indels such as Del_686_9, Del_22281_9, and Del_28248_6 also had negative selection coefficients. The potential reason is epistasis. Research on mutational landscape changes reveals that mutation at the first amino acid may affect the function of another amino acid, which can also be explained by epistasis. Experiments have confirmed the existence of epistasis, such as substitution N501Y (36). Substitution mutations may have epistasis for indels when extended to all mutations. Some models have considered this and made predictions based on the principle of epistasis (37, 38). We think this is worthy of in-depth study.

Although we have identified the relationship between indels and variants and predicted the growth rates of lineages and the selection coefficients of indels, more workers are needed to improve the research on indels. Here our data helped researchers understand the importance of indels during viral evolution, but we should perform the controlled experiment to confirm the evaluation. At the same time, similar research ideas can also be used in researching more epidemic viruses, which may help us understand the evolution of viruses and prevent the occurrence of a pandemic from a broader perspective.

### Conclusions

Millions of deletion and insertion records were detected from 9 million SARS-CoV-2 genomes. Several indel types that have a strong correlation with Pango lineages were found , and 65 indel types were defined as featured indels representing Pango lineages. We found that there were combinations of featured indels among different VOCs and VOIs. We also evaluated the fitness of indels and found that indels also have high fitness as substitution mutations. In conclusion, indels are also important for viral evolution.

## MATERIALS AND METHODS

### Data collection

Genomic sequences and relevant metadata were downloaded from the GISAID website (https://www.gisaid.org/). We downloaded 12,652,854 unaligned sequences with corresponding metadata on August 18th, 2022, and 12,277,968 aligned sequences with problematic sites masked on August 4th, 2022. The phylogenetic tree constructed on July 20th, 2022, with 9,375,971 samples, was also downloaded from GISAID.

### Data preprocess and quality control

Although GISAID allows downloading all SARS-COV-2 sequences, we cannot use these data directly since there are several problems such as duplicated sequences, additional “hCoV-19/” prefixes, and whitespace from strain ids, which can affect the follow-up analysis. To solve those mentioned problems, we used scripts from Nextstrain/ncov to sanitize genomic sequences and metadata (https://github.com/nextstrain/ncov/tree/master/scripts) (39). To correspond with unaligned sequences, aligned sequences, and sequences in the phylogenetic tree, we selected those sequences that existed among unaligned sequences, aligned sequences, and sequences in the phylogenetic tree simultaneously with R packages Tidyverse and SeqKit (40, 41). To control quality, we only selected sequences whose host is human, tagged true for “Is Complete” and false for ”Is low coverage” within the corresponding metadata. Sequences without concrete collection date, location, or Pango lineage were also filtered out. A total of 9,149,680 sequences were finally selected after quality control. The redundant samples in the phylogenetic tree were removed with the R package ape (42).

### Indel identification and analysis

To identify indels from genomic sequences, we used minimap2 to align 9,149,680 filtered sequences to the reference sequence Wuhan-Hu-1 (NC_045512.2) and generated a large SAM file (43). The location and length of indels and insertion sequence were then extracted from the generated SAM file with in-house scripts.

Indels in 5′ and 3′ untranslated regions were filtered out, and other statistics were counted with the R package Tidyverse. Combinations of featured indels were analyzed with the R package ComplexUpset (44, 45). The phylogenetic trees were constructed with FastTree and visualized with the Coronavirus GenBrowser of eGPS (46 – 48).

### Indels refine Pango lineage clusters

Pango lineage clusters have been one of the most widely used taxonomies for COVID-19 in previous works (9). However, it has been proven inappropriate for downstream analyses, such as fitness estimation, due to the severe intralineage inconsistency of fitness (28).

To refine the clusters, Pyro models, which were developed for evaluation of mutations and fitness of lineages, described a procedure consisting of two steps (26):

1. First, an UShER tree (UShER tree was generated with UShER program, which was designed to place new samples onto existing phylogeny rapidly) $T_{0}$ is constructed, where each node corresponds to a genomic sequence (49). An attractive property of the constructed UShER tree is that nodes are placed based on genomic similarity, which would appeal to efficient follow-up steps.
2. Second, a greedy algorithm is employed to prune iteratively $T_{0}$ . Ideally, a node in the tree could be considered a single cluster. However, the number of nodes in the complete tree, dubbed ${N(T}_{0})$ , is directly proportional to that of sequences, resulting in a huge number of nodes when there are millions of sequences. Therefore, a post-process is proposed to reduce the tree size, in which the underlying consideration is to minimize the mutation tree distance. Suppose we want to partition these tree nodes into C clusters; then, we iterate $N\left(T_{0}\right)-C$ steps.

Notably, the UShER tree construction and its pruning process merely consider substitution mutations, missing valuable indel information. We modified the above process to assess the selection coefficient of indels and incorporate them into the evaluation of clusters. After constructing $T_{0}$ in Step (1), we modified the mutation tree by adding the indel information originally ignored in UShER (26). The modified tree $T_{0}^{`}$ is then pruned using the algorithm in Step (2), where indel differences are also measured when calculating the tree difference.

Based on the clustering results, a mutation profile matrix $M^{FC}$ with *F* × *C* elements can be computed, where *F* denotes the number of selected alleles. The element in the *i*th row and *j*th column element $M_{ij}^{FC}$ equals 1 if most sequences in clade c share the *i*th mutation, otherwise equals 0. In addition, a count matrix $O^{TPC}$ with dimensions of *T* × *P* × *C* is based on the frequency. *T* and *P* corresponded to the number of time and region bins, respectively, which accounted for the temporal and spatial factors (26 – 28). Until August 18th, 2022, we split the data into 69 bins (*T* = 69) and 1,729 region bins (*P* = 1560). When neglecting the indel, we first followed the setting in and clustered the sequences into 3,000 clusters (*C* = 3000) with 3,187 mutations (F = 3187) (27, 28). We fixed the pruned loss, added indel information, and then repeated the tree-pruning process. Since the indels improved the pruned loss, we obtained 3,092 clusters (*C* = 3092) with 3,366 mutations (*F* = 3366). Based on these data, experiments were conducted to reveal how indel information impacted the clustering process and fitness estimation.

### Viral fitness assessment

As discussed in references (26 – 28), a discrete-time branching process is adopted to model the process of viral infection. In this case, the infected population of variant *v* at period *t* + 1, dubbed $N_{t+1}(v)$ , could be estimated from that at period *t*, dubbed $N_{t}(v)$ . Considering super spreading, in which a minority of people are responsible for a majority of infected cases, a negative binomial distribution is commonly employed:


$$N_{\left(t+1\right)}\left(v\right)∼NBD\left(r_{t}\left(v\right),p_{t}\left(v\right)\right)$$

where $r_{t}(v)$ and $p_{t}(v)$ represent the expectation and dispersion of NBD, respectively. Given the effective reproduction number of variant v R(v) , as presented in reference (28):


$$\;r_{t}\left(v\right)=N_{t}\left(v\right)\times \;R\left(v\right),\;p_{t}\left(v\right)=R\left(v\right)+R\left(v\right)/k$$

Conventionally, each site *i* in the genome sequence is considered an allele, in which the value is either 0, representing wild type or 1, implying mutations. Here, Wuhan-Hu-1 is considered as a reference, and we then computed the relative fitness *f*(*v*) for each variant *v*. By characterizing each variant *v* with *M* alleles, the relative growth rate of *v*, dubbed *f*(*v*) could be described by an additive model:


$$f\left(v\right)=\sum_{m=1}^{M}F_{v,m}s_{m}$$

where $F_{v,m}$ and $s_{m}$ represent the feature of *v* at the $m_{th}$ allele and the variant selection coefficient of the $m_{th}$ allele, respectively.

We generally followed the implementation in BVAS to estimate the variant fitness and the selection coefficient. The count matrix $O^{TPC}$ and mutation profile matrix $M^{FC}$ are input to the modified BVAS model to estimate the fitness of clusters and the PIP scores of specific mutations. While the original BVAS ignores the indel information, we showed that by including this information, the BVAS could better predict fitness. Furthermore, the modified BVAS showed that several indels have high selection coefficients and PIP scores, which emphasized indels’ role in viral transition and evolution.

### Simulation

Simulation experiments are also performed to verify the performance of our pipeline to estimate the selection coefficients. Specifically, genomes are composed of a set of alleles, of which parts are assumed to be neutral, while the others are causal. The true selection coefficients of neutral alleles are set to zero, while causal alleles are set to a number between [−0.1 and 0.1], as in BVAS. Pandemic data are then generated following a non-negative binomial distribution. Based on the generated data, BVAS estimation is employed to estimate the selection coefficient. Finally, precision and recall are used to measure the estimation performance. Precision is calculated by the number of truly identified alleles divided by that of causal alleles, while recall is defined as the truly identified causal alleles divided by that of the true causal alleles.

For hyperparameters, we used a similar setting in BVAS. A total of 100 alleles are considered in our experiments. For neutral alleles, the true selection coefficients are set to zero, and those of causal alleles are randomly set to [0.01, 0.02, 0.04, 0.06, 0.08,–0.01, -0.02,–0.04, -0.06,–0.08]. The number of causal alleles is increased from 10 to 50, with a step of 10. With each experiment repeated 200 times, a total of 200 times, 1,000 experiments are performed. The estimated positives are those with a PIP score no smaller than 0.1 (Fig. 7D; Table S15).

## Data Availability

All source codes are based on either Python (version 3.8) or R (version 4.2) and can be found in the GitHub repository. All supplementary files, including figures and tables, are at figshare.
